# Donut rush to laparoscopy: post-polypectomy electrocoagulation syndrome and the ‘pseudo-donut’ sign

**DOI:** 10.1259/bjrcr.20190023

**Published:** 2020-09-29

**Authors:** Isabella Plumptre, Timo Tolppa, Zaynab A.R. Jawad, Noman Zafar

**Affiliations:** 1 Department of General Surgery, Northwick Park Hospital, London North West University Healthcare NHS Trust, London, UK

## Abstract

Colonoscopic polypectomy is a routine procedure with the potential for rare but well-known complications, including perforation and bleeding. Post-polypectomy electrocoagulation syndrome (PPES) is a less recognized cause of abdominal pain following this procedure. However, it is important to diagnose PPES in order to avoid unnecessary intervention. We present the case of a patient with abdominal pain after polypectomy. The patient underwent an unnecessary diagnostic laparoscopy on the basis of misinterpreted radiological findings. Her CT scan demonstrated the "donut" sign that was suggestive of ileocaecal intussusception. This case highlights the importance of recognizing PPES as a possible cause for abdominal pain after colonoscopic polypectomy and that it may also present with a "pseudodonut" sign on CT scan. It also demonstrates the importance of communicating and then integrating full clinical details with radiological findings when formulating a differential diagnosis.

## Case presentation

A 64-year-old female presented to the Emergency Department with generalized abdominal pain associated with nausea and diarrhoea. On the morning of presentation, the patient had undergone a colonoscopy during which 4 polyps, up to 18 mm in size, were removed from the caecum. Her pain had started a few hours after the procedure. She denied any fever, vomiting or bleeding per rectum. Her past medical history included dysfibrinogenaemia and previous laparoscopic cholecystectomy. On examination, she was afebrile and haemodynamically stable. She was found to have generalized abdominal tenderness worst in the right iliac fossa and associated with localized guarding. Her blood tests showed a slightly elevated white cell count of 11.1 × 10^9/L and raised C-reactive protein of 161 mg l^−1^. Her renal function was normal. Her abdominal radiograph was also normal.

**Figure 1.**
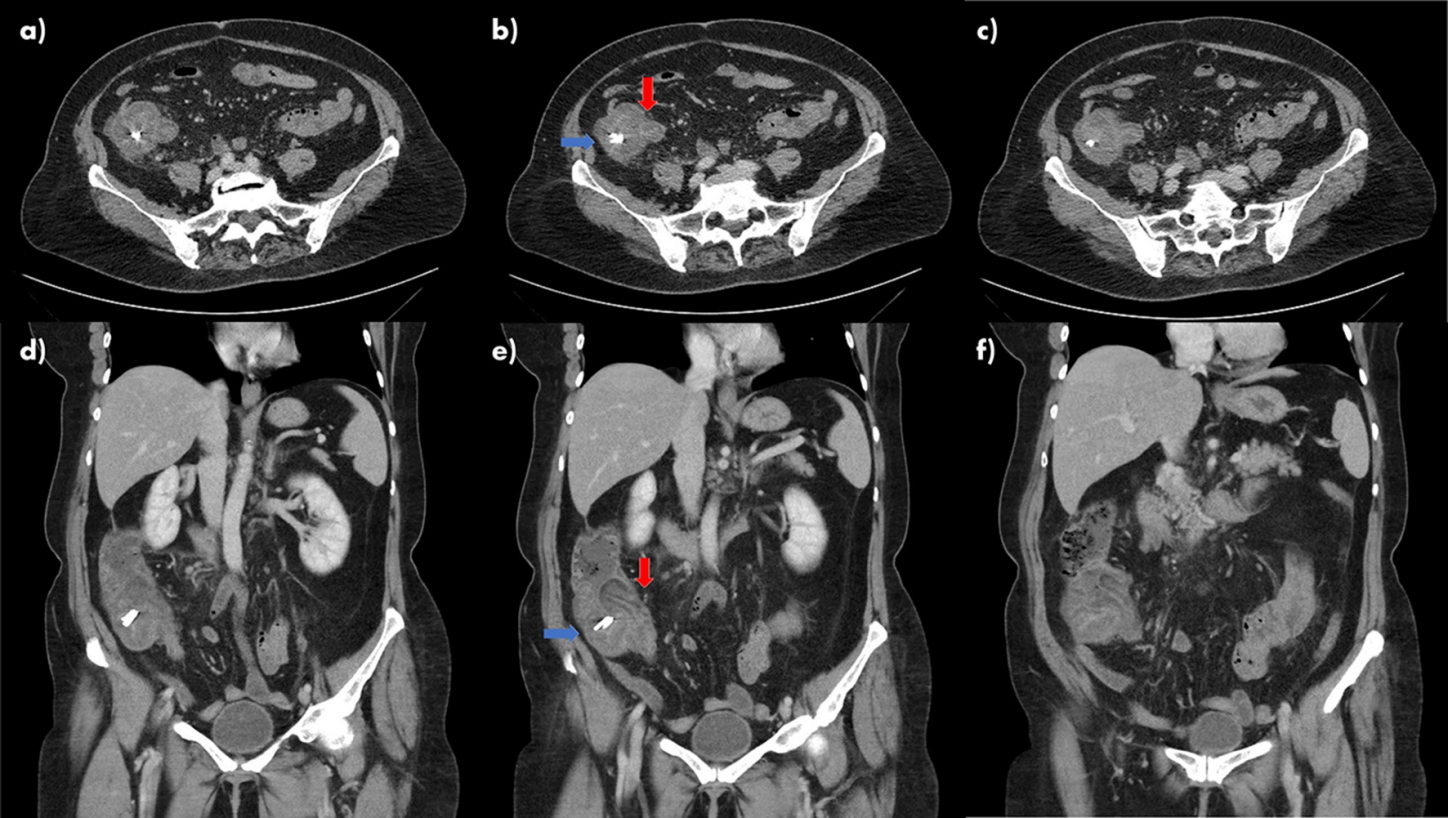
(a–f) Biphasic contrast-enhanced CT scan of the abdomen and pelvis demonstrating the "pseudodonut" sign as a result of thickening of ileocaecal wall due to post-polypectomy syndrome in successive axial (a–c) and coronal (d–f) images. Blue arrows (b, e) show caecum containing radiopaque clips applied during colonoscopy. Red arrows (b, e) show the ileocaecal valve. The thickening of the terminal ileum gives the impression of the donut sign seen in intussusception.

A biphasic CT scan of the abdomen and pelvis with contrast was performed. The CT scan was provisionally reported as showing thickening of the wall of the caecum and terminal ileum with surrounding inflammatory changes consistent with colitis. It also demonstrated a filling defect at the junction of the portal and splenic veins consistent with an acute thrombus. However, the final report of the CT scan disagreed with this and reported ileocolic intussusception. This report was based on images showing a 4 cm invagination of the terminal ileum into the caecum without proximal bowel dilatation (Figure 1).

The patient was initially managed conservatively for suspected post-polypectomy electrocoagulation syndrome (PPES). However, 24 h later, in the context of worsening clinical signs and the final radiology report suggesting intussusception, the patient underwent a diagnostic laparoscopy. Induction of general anaesthesia was unexpectedly complicated. The patient was found to have a difficult airway which led to a prolonged anaesthetic time. Following unsuccessful mask ventilation, she required direct laryngoscopic intubation for a high Grade III airway. Intraoperatively, the caecum was inflamed with no evidence of intussusception or perforation.

Postoperatively, the conflict of radiology reports and intraoperative findings were discussed with the patient. In view of the history of dysfibrinogenaemia and splenic vein thrombosis, the patient was started on anticoagulation prior to discharge on the advice of the haematologists. Following this, the patient made an excellent recovery.

## Discussion

Polypectomy is a common and low-risk procedure carried out during colonoscopy with the aim of reducing the incidence of colorectal cancer by removing pre-cancerous dysplastic lesions. Well-recognized complications of polypectomy include perforation and bleeding, and though they can be life-threatening they are relatively rare in practice.^1^ However, despite their rarity, it is important to exclude these complications in a patient presenting shortly following the procedure. In the case presented herein, the history of localized abdominal pain associated with tenderness and guarding as well as raised inflammatory markers within 24 h of a polypectomy may also be compatible with PPES. CT scan is recommended in these patients to exclude the more serious complication of bowel perforation.^1^

PPES, otherwise known as transmural burn syndrome or microperforation, is caused by an injury to the colonic mucosa and muscularis layer, resulting in peritoneal inflammation.^1,2^ The incidence of PPES ranges from 0.003 to 0.1%, whilst the rates of perforation and bleeding are 0.3 and 0.6% respectively.^2^ PPES characteristically presents within 12 h of colonoscopy with localized abdominal pain at the site of the polypectomy, fever, peritoneal inflammation and elevated inflammatory markers.^1,2^ Apart from fever, this case had a typical presentation of PPES. Additionally, the patient was at a higher risk of an adverse event based on the size of the polyps. Patients undergoing polypectomy for polyps larger than 1.5 cm, as well as those located in the caecum, are more prone to complications following polypectomy.^3^

The signs and symptoms of PPES mimic the clinical presentation of a perforation. Differentiation from perforation is essential in order to prevent an unnecessary exploratory laparotomy.^1,2^ PPES commonly resolves with conservative management including intravenous fluids, antibiotics and by keeping the patient nil by mouth. On the other hand, a perforation may require surgery.^2^ The gold-standard investigation for patients presenting in this way is a CT scan, which in the case of PPES demonstrates focal mural thickening with a stratified enhancement pattern, low attenuation perilesional submucosal oedema, and high attenuation infiltration of adjacent pericolonic fat in the absence of extraluminal air.^4^ In the case of this patient, the CT scan was able to differentiate between perforation and PPES. However, the CT scan raised a diagnostic dilemma due to the interpretation of a "donut sign" suggestive of intussusception.

In view of the report, a decision was made by the surgical team to proceed with a diagnostic laparoscopy and the patient was therefore unnecessarily exposed to the possible complications of the operation. The risks of the operation were increased for our patient due to the pre-existing hypercoagulable state from untreated dysfibrinogenaemia. Additionally, our patient had a difficult intubation, which could have resulted in further complications. The diagnostic laparoscopy could have been avoided with careful consideration of the likely cause of the patient’s symptoms and taking into account the clinical history. Furthermore, awareness that PPES is a possible complication of polypectomy and that this may also manifest on CT scan with a "pseudo donut" sign may have also avoided an unnecessary procedure. In view of the recent polypectomy, relevant risk factors, characteristic symptoms and signs, and the CT scan findings, the presentation of the patient was most consistent with PPES. Conversely, ileocaecal intussusception in adults is more likely to cause vomiting and is associated with tumours in 93.8% of cases, making the diagnosis in this case unlikely.^5^ This case demonstrates the importance of combining the clinical scenario with the radiological investigations in order to achieve a differential diagnosis and correct management plan.

## Conclusion

PPES is a less well-known complication that should be considered in patients less than 12 h post-polypectomy, presenting with fever, localized peritonitis and raised inflammatory markers. Surgeons and radiologists should be aware of the "pseudodonut" sign and balance radiological findings in the full context of the patient’s presentation before considering surgical intervention.

## Learning points

- Consider PPES in the differential diagnosis in febrile patients with local peritonitis and raised inflammatory markers presenting within 12 h of polypectomy.
- PPES may present with a "pseudodonut" sign on abdominal imaging.
- Although management of PPES is generally conservative, it is important to recognize promptly in order to avoid unnecessary surgical procedures.
